# Application of cellular microstructural diffusion MRI (cell size imaging) in rectal lesions: a preliminary study

**DOI:** 10.3389/fonc.2025.1535271

**Published:** 2025-02-03

**Authors:** Peisi Kou, Liangjie Lin, Ying Li, Hui Qin, Kun Zhang, Wenhua Zhang, Juan Li, Yong Zhang, Jingliang Cheng

**Affiliations:** ^1^ Department of Magnetic Resonance Imaging (MRI), The First Affiliated Hospital of Zhengzhou University, Zhengzhou, China; ^2^ Clinical and Technical Support, Philips Healthcare, Beijing, China; ^3^ Department of Pathology, The First Affiliated Hospital of Zhengzhou University, Zhengzhou, China

**Keywords:** rectal lesion, adenocarcinoma, magnetic resonance imaging, diffusion, microstructure

## Abstract

**Objectives:**

To explore the value of cellular microstructural mapping by IMPULSED (imaging microstructural parameters using limited spectrally edited diffusion) method in evaluating the histological type and prognostic factors of rectal lesions.

**Materials and methods:**

Sixty-six patients with rectal lesions were enrolled in this study. All subjects underwent MRI scans including conventional diffusion weighted imaging (DWI) and the IMPULSED MRI scans of oscillating gradient spin-echo (OGSE) and pulse gradient spin-echo (PGSE) sequences. Parameters including mean cell diameter (d_mean_), intracellular fraction (v_in_), extracellular diffusivity (d_ex_), cellularity, and apparent diffusion coefficient (ADC) values (ADC_PGSE_, ADC_17Hz_, ADC_33Hz_, and ADC of conventional DWI) were measured in different histopathologic types, grades, stages, and structure invasion statuses. The receiver operating characteristic (ROC) curve analysis was used to evaluate diagnostic power. The sensitivity, specificity, and the corresponding area under the curves (AUCs) were calculated.

**Results:**

Our preliminary results illustrated that malignant lesion showed higher v_in_ and cellularity ([0.2867 ± 0.0697] vs. [0.1856 ± 0.1011], [2.3508 ± 0.6055] vs. [1.2716 ± 0.4574], all *P*<0.05), lower d_ex_ and ADC values (ADC_PGSE_, ADC_17Hz_, and ADC of conventional DWI) compared to benign lesion ([2.1637 ± 0.3303 μm^2^/ms] vs. [2.5595 ± 0.5085 μm^2^/ms], [0.9238 (0.7959, 1.0741) ×10^-3^ mm^2^/s] vs. [1.3373 ± 0.3902×10^-3^ mm^2^/s], [1.3204 ± 0.2342×10^-3^ mm^2^/s] vs. [1.8029 ± 0.3119×10^-3^ mm^2^/s], [0.7400 (0.6750, 0.8375) ×10^-3^ mm^2^/s] vs. [1.0550 ± 1.1191×10^-3^ mm^2^/s], all *P*<0.05), while no significant difference was seen for d_mean_. V_in_ and cellularity of rectal common adenocarcinoma (AC) were significantly higher than those of rectal mucinous adenocarcinoma (MC) ([0.2994 ± 0.0626] vs. [0.2028 ± 0.0571], [2.4579 ± 0.5553] vs. [1.6412 ± 0.4347], all *P*<0.05), while dex and ADC values (ADC_PGSE_, ADC_17Hz_, ADC_33Hz_, and ADC of conventional DWI) were lower in AC ([2.1189 ± 0.3187 μm^2^/ms] vs. [2.4609 ± 0.2534 μm^2^/ms], [0.8996 ± 0.1583×10^-3^ mm^2^/s] vs. [1.2072 ± 0.2326×10^-3^ mm^2^/s], [1.2714 ± 0.1916×10^-3^ mm^2^/s] vs. [1.6451 ± 0.2420×10^-3^ mm^2^/s], [1.8963 (1.6481, 2.1138) ×10^-3^ mm^2^/s] vs. [2.3104 ± 0.3851×10^-3^ mm^2^/s], [0.7341 ± 0.8872×10^-3^ mm^2^/s] vs. [1.1410 ± 0.1840×10^-3^ mm^2^/s], all *P*<0.05). In AC group, the d_mean_ had significant difference between negative and positive tumor budding (TB) ([13.2590 ± 1.3255 μm] vs. [14.3014 ± 1.1830 μm], *P*<0.05). No significant difference of d_mean_, v_in_, d_ex_, cellularity or ADC values was observed in AC with different grade, T stage, N stage, perineural and lymphovascular invasion (all *P*>0.05). The ROC curves showed that the area under the curves (AUCs) of v_in_, d_ex_, cellularity, and ADC values (ADC_PGSE_, ADC_17Hz_, and ADC of conventional DWI) for distinguishing malignant and benign lesion were 0.803, 0.757, 0.948, 0.807, 0.908 and 0.905, respectively. The AUCs of v_in_, d_ex_, cellularity, and ADC values (ADC_PGSE_, ADC_17Hz_, ADC_33Hz_, and ADC of conventional DWI) in distinguishing AC from MC were 0.887, 0.802, 0.906, 0.896, 0.896, 0.781 and 0.991, respectively. The AUC of the d_mean_ for evaluating TB status was 0.726. The AUC of ADC from conventional DWI for evaluating WHO grade was 0.739.

**Conclusion:**

Cellular microstructural mapping by the IMPULSED method has great potential in preoperative evaluation of rectal lesions. It could be helpful in differentiating malignant and benign lesions, distinguishing AC from MC, and in predicting the TB status.

## 1 Introduction

Colorectal cancer (CRC) is a common malignant tumor in the digestive system, which is the third most common cancer and the second most common cause of cancer-related death in global (1). About 30% occur in the rectum (2), and 90% are classified as adenocarcinoma (AC). Many factors affect the choice of treatment methods and prognosis of patients, such as pathological types, tumor grade, T stage, and N stage. For example, rectal mucinous adenocarcinoma (MC) is a common subtype of rectal adenocarcinomas, which is not sensitive to neoadjuvant chemoradiotherapy and has a poor prognosis. In addition to malignant tumors, benign lesions could also occur in the rectum, such as rectal adenomas, inflammatory lesions, etc. However, preoperative classification of rectal lesions remains challenging. Magnetic resonance imaging (MRI) has become the preferred imaging method for diagnosis of rectal lesions due to its excellent soft tissue resolution. However, few evidences are available about the application of MRI in preoperative evaluation of rectal benign lesions.

The conventional diffusion-weighted magnetic resonance imaging (dMRI) along with the derived apparent diffusion coefficient (ADC) has shown important diagnostic value in rectal lesions (3–6). Nevertheless, its performance in rectal cancer subtype analyses and the evaluation of prognostic factors is limited. One of the key reasons may lie in that ADC is a general measurement of restricted diffusion rate that cannot pinpoint the underlying pathology. Micro cellular structures including cell size, cell density, and intra- or extra-cellular volume fractions could change ADC. Recent advances in diffusion MRI for microstructural modeling provide the opportunity to characterize cancer pathology *in vivo* (7). Among them, the IMPULSED (imaging microstructural parameters using limited spectrally edited diffusion) method developed by Jiang etc. has been used in the study of multiple systemic diseases in patients with breast, prostate cancer, and brain tumor (8–11). However, the application value of IMPULSED MRI in evaluating pathological features of rectal lesions remains unclear.

The current study aims to explore the efficacy of the IMPULSED MRI for microstructural mapping in rectal lesions, and further to evaluate whether the obtained tumor microstructural properties could be used to distinguish prognostic factors in rectal cancer.

## 2 Materials and methods

### 2.1 Study participants

**Figure 1:**
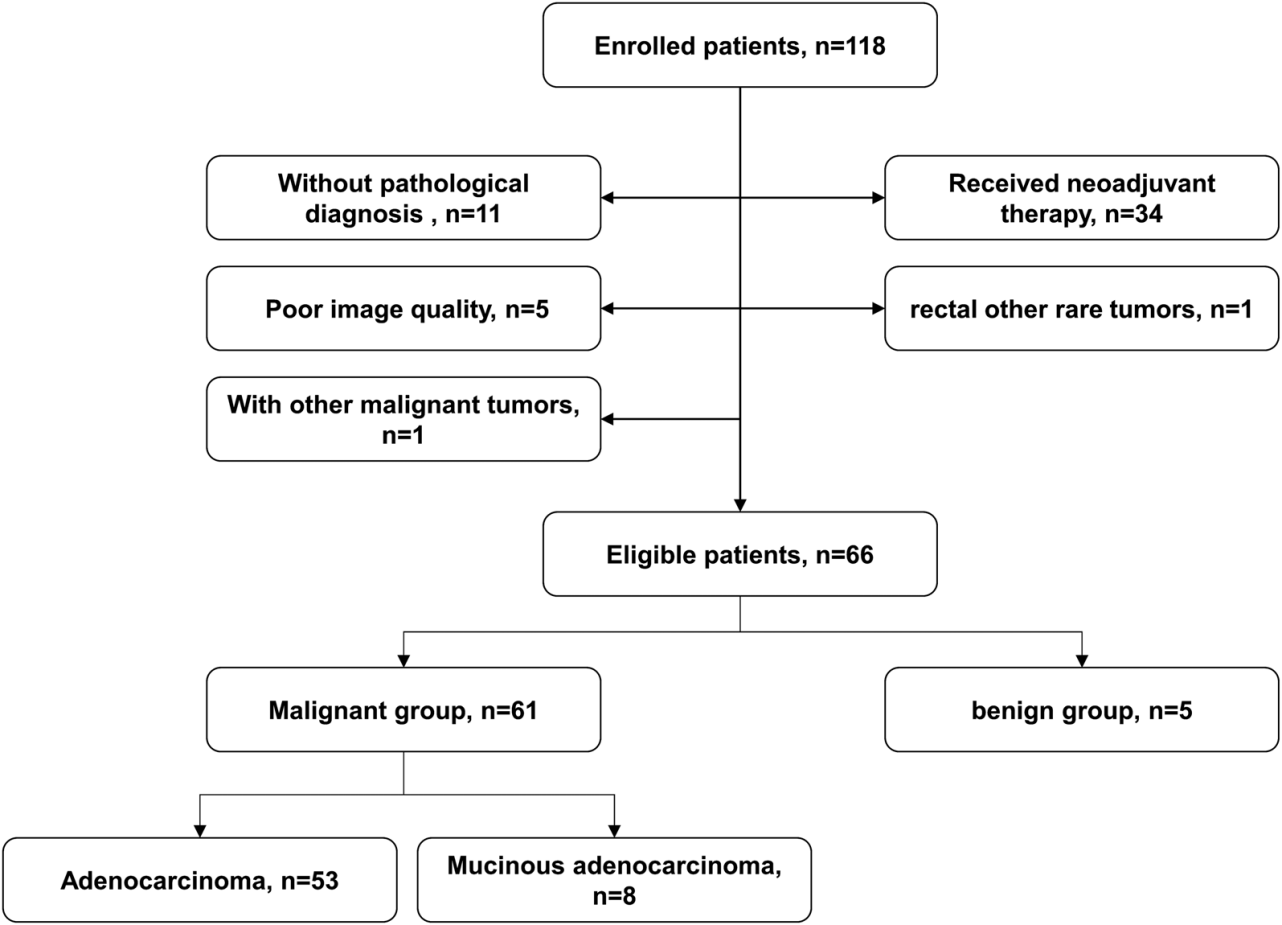
Flowchart of patient enrolment.

This preoperative study included 118 patients who were diagnosed with rectal lesions between April 2023 and September 2023 at our hospital. The inclusion criteria were as follows: (1) patients suspected of rectal lesions; (2) patients without surgery, chemoradiotherapy or other related treatments before the MRI examination; (3) pathological diagnosis could be obtained within two weeks after the MRI examination; The exclusion criteria were as follows: (1) patients without pathological diagnosis (n=11); (2) patients received neoadjuvant therapy (n=34); (3) poor image quality (n=5); (4) patients with rectal lymphomas, neuroendocrine tumor and other rare tumors (n=1); and (5) patients with a history of other malignant tumors (n=1). Finally, 66 patients were enrolled in this study cohort (**Figure 1**).

### 2.2 Magnetic resonance imaging protocols

MRI was performed on a 3T scanner (Ingenia Elition, Philips Healthcare, Best, the Netherlands) with a high-performance gradient system (maximum gradient = 45 mT/m per axis, maximum slew rate = 220 mT/m) and a 24-channel abdominal coil. Patients need to empty the rectum before MRI examination. The oscillating gradient spin-echo (OGSE) sequence with trapezoid-cosine gradients and the pulse gradient spin-echo (PGSE) diffusion weighted sequence were implemented with 2D echo-planar imaging acquisition. **Table 1** shows detailed diffusion encoding parameters for PGSE and OGSE scans. Routine pre-contrast MRI scans included T1-weighted imaging, fat-suppressed T2-weighted imaging, and conventional diffusion-weighted imaging (DWI). The post-contrast T1-weighted images were acquired for anatomical reference. The patients did not require too much special preparations, and the whole scan took less than half an hour.

**Table 1:** Scan parameters for the IMPULSED MRI of PGSE and OGSE sequences.

	PGSE	OGSE_17Hz_	OGSE_33Hz_
TR (ms)	4000	4000	4000
TE (ms)	145	145	145
Field of views (mm^2^)	192×192	192×192	192×192
Voxel size (mm^3^)	2.53×2.58×5	2.53×2.58×5	2.53×2.58×5
Flip angle (°)	90	90	90
Matrix size	76×74	76×74	76×74
Reconstructed voxel size (mm^2^)	1.2×1.2×5M	1.2×1.2×5	1.2×1.2×5
Cycle	/	1	2
f (Hz)	/	17	33
Effective td (ms)	26.7	15	7.5
b-value (s/mm^2)^	0/250/500/750/1000/1400/1800	0/250/500/750/1000	0/100/200/300
Bandwidth (pixel/Hz)	37.2	37.2	37.2
Scan duration	4min24s	4min12s	2min08s

IMPULSED, imaging microstructural parameters using limited spectrally edited diffusion; MRI, magnetic resonance imaging; PGSE, pulse gradient spin-echo; OGSE, oscillating gradient spin-echo.

### 2.3 Image analysis and data acquisition

High-resolution T2WI was used to evaluate tumor location, tumor size, and bowel circumferential involvement. The estimation of IMPULSED parameters included mean cell diameter (d_mean_), intracellular fraction (v_in_), extracellular diffusivity (d_ex_), and cellularity, while intracellular diffusivity (D_in_) was fixed at 1.58 μm^2^/ms to ensure fitting stability. The parameters were constrained to 4 < d_mean_ <30 μm, 0 < v_in_ < 1, and 0 < d_ex_ <3.5 μm^2^/ms based on physiologically relevant values. The fitting was performed using the least square curve fitting toolbox in MATLAB (Mathworks, Inc.). Additionally, the apparent diffusion coefficient (ADC) values were fitted according to S/S0 = exp(−b×ADC) using a log-linear fitting with all b values for PGSE/OGSE.

In patients with rectal lesion, the regions-of-interest (ROIs) were manually delineated on the slice with the largest scale of the lesion based on diffusion-weighted images by two experienced radiologists independently, and necrotic area or surrounding tissue was carefully excluded from the segmentation. The fitted microstructural parameters, including d_mean_, v_in_, d_ex_, cellularity, and ADC (ADC_PGSE_, ADC_17Hz_, ADC_33Hz_), were calculated in a voxel-wise manner and averaged within the tumor ROIs. And the image quality was evaluated by two radiologists independently according to the artifact and noise (1 score, both strongly obvious; 2 score, both obvious; 3 score, slight artifacts and noise, but acceptable; 4 score, no artifact and slight noise; 5 score, no artifact and noise).

### 2.4 Pathologic evaluation

The pathological diagnosis was evaluated by a pathologist with >5 years of experience, who was blinded to the MRI diagnosis. The pathological reports included the diagnosis of benign and malignant lesions. For malignant lesions, pathological reports should contain histological types, tumor grade, pathological stage, perineural invasion (PNI), and lymphovascular invasion (LVI), and tumor budding (TB). Tumor grades were classified as low-grade and high-grade, the former included grade 1 (G1, well- differentiated, >95% gland forming) and grade 2 (G2, moderately differentiated, 50–95% gland forming), and the latter referred to grade 3 (G3, poorly differentiated, 0-49% gland forming). Pathological stage included T staging and N staging. Pathological T staging was divided into early and late stages according to the American Joint Committee on Cancer (AJCC) 8th edition. Early-stage rectal cancer included stages pT1 and pT2, which was defined as tumor confined to the muscularis propria. Late-stage rectal cancer included stages pT3 and pT4, which referred to tumor extending beyond the muscularis propria. According to the status of regional lymph node, pathological N staging was divided into two categories, including lack of regional lymph node metastases (pN0) and regional lymph node metastasis (pN1-2). PNI, LVI and TB statuses were classified into positive and negative groups.

### 2.5 Statistical analysis

SPSS 27.0 software (IBM Corp., Armonk, NY, USA) and MedCalc (MedCalc Software, Mariakerke, Belgium) were used for the statistical analyses. GraphPad Prism software (version 10.1.2, GraphPad Software, San Diego, CA, USA) was used to make diagrams. The Shapiro–Wilk test (n ≤ 50) and Kolmogorov-Smirnov test (n>50) were used to determine normal or skewed data distribution for all continuous variables. The normally distributed data were expressed as mean ± standard deviation (SD). The non-normally distributed data were expressed as median (1st quartile, 3rd quartile). The two-independent samples *t*-test or the Mann–Whitney U test were used to compare d_mean_, v_in_, d_ex_, cellularity and ADC parameters between histological types (malignant lesion vs. benign lesion; AC vs. MC), tumor grades (low grade vs. high grade), pT stages (pT1-2 vs. pT3-4), pN stages (pN0 vs. pN1-2), PNI (negative vs. positive), LVI (negative vs. positive), and TB (negative vs. positive). For parameters with significant differences, the receiver operating characteristic (ROC) curve analysis was used to evaluate diagnostic power. The sensitivity, specificity, and area under the curve (AUC) were calculated, and the cut-off value was also obtained using the Youden index (Youden’s index = sensitivity + specificity - 1). The intraclass correlation coefficient (ICC) and Kappa test were used to evaluate the agreement of measured parameters between the two radiologists. ICC values of greater than 0.75 and Kappa coefficient of greater than 0.80 were considered to good agreement. A probability of *P* values <0.05 was considered statistically significant.

## 3 Results

### 3.1 Patient characteristics

A total of 66 patients (61 in the malignant group and 5 in the benign group) were enrolled in this study. In the malignant group, 53 were classified as AC and 8 were classified as MC according to the results of postoperative pathology. Basic demographic and clinical information of the patients were summarized in **Table 2**.

**Table 2:** Clinical and pathologic characteristics of the study patients.

Characteristics	Number of patients
Gender	
Male	41
Femal	25
Age	
Mean age, years	59 ± 9.322
Age range, years	37-78
Pathological type	
benign lesions	5
MC	8
AC	53
WHO grade (AC)	
Low-grade	39
High-grade	14
T stage (AC)	
pT1-2	19
pT3-4	34
N stage (AC)	
pN0	23
pN1-2	30
PNI (AC)	
Negative	27
Positive	26
LVI (AC)	
Negative	29
Positive	24
TB (AC)	
Negative	24
Positive	29

MC, mucinous adenocarcinoma; AC, adenocarcinoma; PNI, perineural invasion; LVI, lymphovascular invasion; TB, tumor budding.

### 3.2 Agreement of measured parameters between the two radiologists

There were good agreements between two observers. The ICC were 0.879 (95% CI 0.810–0.924) for d_mean_; 0.909 (95% CI, 0.849–0.945) for v_in_; 0.889 (95%CI, 0.812–0.933) for d_ex_; 0.863 (95% CI,0.785–0.914) for cellularity; 0.914 (95% CI, 0.863–0.947) for ADC_PGSE_, 0.929 (95% CI, 0.887–0.956) for ADC_17Hz_, 0.924 (95% CI, 0.879–0.953) for ADC_33Hz_, and 0.974 (95% CI, 0.958-0.984) for ADC of conventional DWI, respectively. In addition, it showed high consistency in the evaluation of image quality (κ = 0.813).

### 3.3 Microstructural features and ADCs of different pathological types in rectal lesion

**Figure 2:**
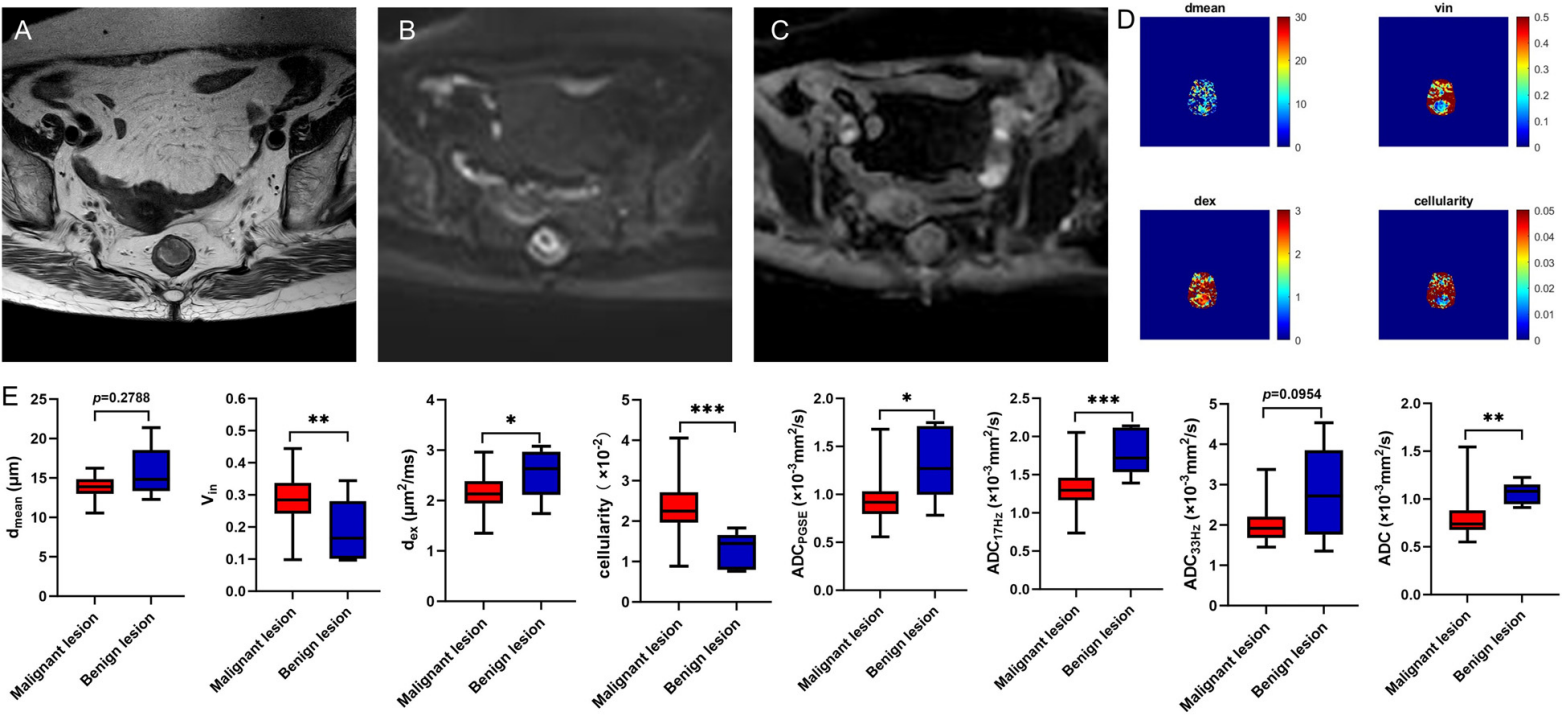
A 78-year-old female with rectal adenoma. Oblique axial T2WI shows a mass with a slight high-intensity signal in the rectum **(A)**; The mass showed high signal on DWI **(B)** and slightly low-intensity signal on ADC map **(C)**; The d_mean_, v_in_, d_ex_, cellularity and ADC values (ADC_PGSE_, ADC_17Hz_, and ADC_33Hz_) were 12.2847 μm, 0.1650, 2.4879 μm^2^/ms, 1.4459×10^-2^, 1.2696×10^-3^ mm^2^/s, 1.7180×10^-3^ mm^2^/s, and 2.1767×10^-3^ mm^2^/s, respectively **(D)**. Box plots of d_mean_, v_in_, d_ex_, cellularity and ADC values (ADC_PGSE_, ADC_17Hz_, ADC_33Hz_, and ADC of conventional DWI) between malignant and benign lesions **(E)**. T2WI, T2 weighted imaging; DWI, diffusion weighted imaging; ADC, apparent diffusion coefficient; *P < 0.05; **P < 0.01; ***P < 0.001.

**Figure 3:**
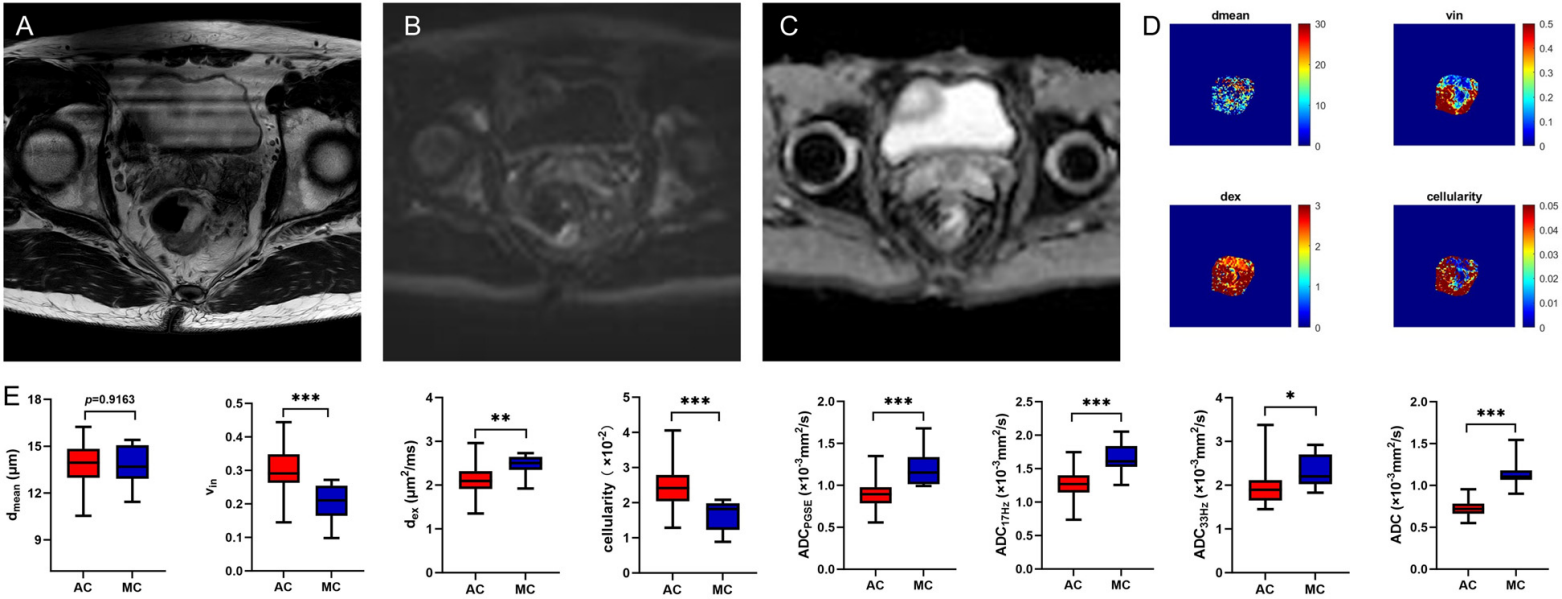
A 59-year-old male with MC. Oblique axial T2WI shows a mass with a high-intensity signal in the rectum **(A)**; The mass showed isointensity signals on DWI **(B)** and high signal on ADC map **(C)**; The d_mean_, v_in_, d_ex_, cellularity and ADC values (ADC_PGSE_, ADC_17Hz_, and ADC_33Hz_) were 14.5828 μm, 0.2628, 2.5125 μm^2^/ms, 2.0014×10^-2^, 1.0622×10^-3^ mm^2^/s, 1.5864×10^-3^ mm^2^/s, and 2.1282×10^-3^ mm^2^/s, respectively **(D)**. Box plots of d_mean_, v_in_, d_ex_, cellularity and ADC values (ADC_PGSE_, ADC_17Hz_, ADC_33Hz_, and ADC of conventional DWI) between AC and MC **(E)**. AC, adenocarcinoma; MC, mucinous adenocarcinoma; T2WI, T2 weighted imaging; DWI, diffusion weighted imaging; ADC, apparent diffusion coefficient; *P < 0.05; **P < 0.01; ***P < 0.001.

Compared to benign lesion, the v_in_ and cellularity were significantly higher, the d_ex_ and ADC values (ADC_PGSE_, ADC_17Hz_, and ADC of conventional DWI) were significantly lower in malignant lesion (all *P*<0.05) (**Table 3**, **Figure 2**). There was no significant difference about d_mean_ between the two groups. V_in_ and cellularity of rectal common adenocarcinoma (AC) were significantly higher than those of rectal mucinous adenocarcinoma (MC), while d_ex_ and ADC values (ADC_PGSE_, ADC_17Hz_, ADC_33Hz_, and ADC of conventional DWI) were lower in AC (all *P*<0.05) (**Table 4**, **Figure 3**).

**Table 3:** Comparison of d_mean_, v_in_, d_ex_, cellularity and ADC values (ADC_PGSE_, ADC_17Hz_, ADC_33Hz_, and ADC of conventional DWI) between malignant and benign lesions.

Pathologic type	d_mean_ (μm)	v_in_	d_ex_ (μm^2^/ms)	cellularity (×10^-2^)	ADC_PGSE_ (×10^-3^ mm^2^/s)	ADC_17Hz_ (×10^-3^ mm^2^/s)	ADC_33Hz_ (×10^-3^ mm^2^/s)	ADC (×10^-3^ mm^2^/s)
Malignant lesion (n=61)	13.8106 ± 1.3289	0.2867 ± 0.0697	2.1637 ± 0.3303	2.3508 ± 0.6055	0.9238 (0.7959, 1.0741)	1.3204 ± 0.2342	1.9376 (1.6861, 2.2621)	0.7400 (0.6750, 0.8375)
Benign lesion (n=5)	15.7237 ± 3.4089	0.1856 ± 0.1011	2.5595 ± 0.5085	1.2716 ± 0.4574	1.3373 ± 0.3902	1.8029 ± 0.3119	2.7902 ± 1.1875	1.0550 ± 1.1191
*P* value	0.2788	0.0037	0.0161	0.0002	0.0208	0.0001	0.0954	0.003

ADC, apparent diffusion coefficient.

**Table 4:** Comparison of d_mean_, v_in_, d_ex_, cellularity and ADC values (ADC_PGSE_, ADC_17Hz_, ADC_33Hz_, and ADC of conventional DWI) between AC and MC.

Malignant lesion	d_mean_ (μm)	v_in_	d_ex_ (μm^2^/ms)	cellularity (×10^-2^)	ADC_PGSE_ (×10^-3^ mm^2^/s)	ADC_17Hz_ (×10^-3^ mm^2^/s)	ADC_33Hz_ (×10^-3^ mm^2^/s)	ADC (×10^-3^ mm^2^/s)
AC (n=53)	13.8177 ± 1.3431	0.2994 ± 0.0626	2.1189 ± 0.3187	2.4579 ± 0.5553	0.8996 ± 0.1583	1.2714 ± 0.1916	1.8963 (1.6481, 2.1138)	0.7341 ± 0.8872
MC (n=8)	13.7640 ± 1.3170	0.2028 ± 0.0571	2.4609 ± 0.2534	1.6412 ± 0.4347	1.2072 ± 0.2326	1.6451 ± 0.2420	2.3104 ± 0.3851	1.1410 ± 0.1840
*P* value	0.9163	0.0001	0.0053	0.0002	0.0000	0.0000	0.011	0.0000

ADC, apparent diffusion coefficient; AC, adenocarcinoma; MC, mucinous adenocarcinoma.

### 3.4 Comparison of microstructural features and ADCs in different subtype groups of rectal adenocarcinomas

**Figure 4:**
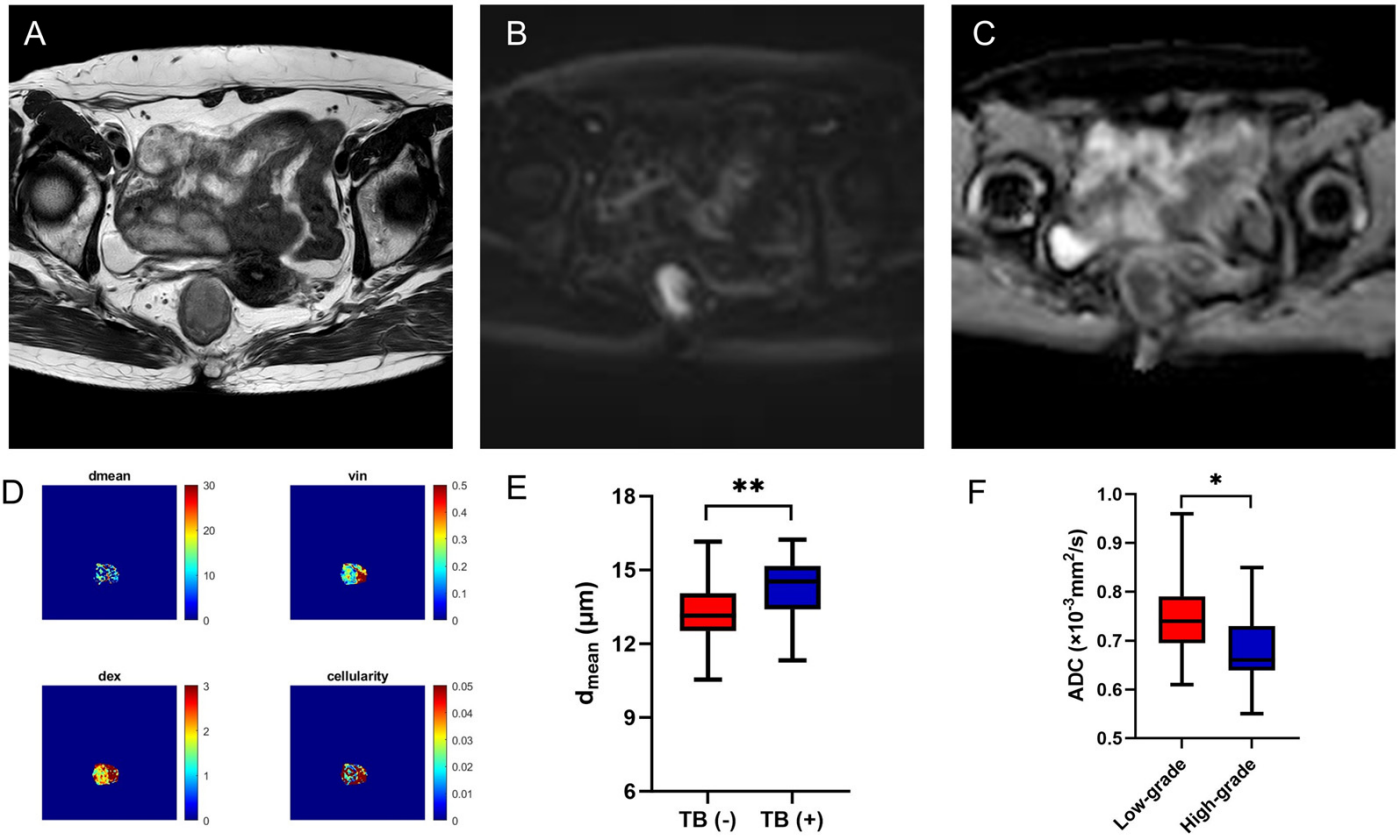
A 56-year-old female with AC. Oblique axial T2WI shows a mass with a slight high-intensity signal in the rectum **(A)**; The mass showed high signal on DWI **(B)** and obviously low-intensity signal on ADC map **(C)**; The d_mean_, v_in_, d_ex_, cellularity and ADC values (ADC_PGSE_, ADC_17Hz_, and ADC_33Hz_) were 14.0769 μm, 0.2781, 1.8814 μm^2^/ms, 2.1481×10^-2^, 0.8677×10^-3^ mm^2^/s, 1.2180×10^-3^ mm^2^/s, and 1.7120×10^-3^ mm^2^/s, respectively **(D)**. Box plots of d_mean_ between negative and positive TB of AC **(E)**. Box plots of ADC of conventional DWI between low-grade and high-grade of AC **(F)**. AC, adenocarcinoma; T2WI, T2 weighted imaging; DWI, diffusion weighted imaging; ADC, apparent diffusion coefficient; TB, tumor budding; *P < 0.05; **P < 0.01

In AC group, the d_mean_ was higher in positive tumor budding (TB) group (*P*<0.05), and ADC of conventional DWI was higher in low-grade group (*P*<0.05) (**Table 5**, **Figure 4**). No significant difference of d_mean_, v_in_, d_ex_, cellularity or other ADC values (ADC_PGSE_, ADC_17Hz_, ADC_33Hz_, and ADC of conventional DWI) was observed in other groups (all *P*>0.05).

**Table 5:** The comparison of d_mean_, v_in_, d_ex_, cellularity and ADC values (ADC_PGSE_, ADC_17Hz_, ADC_33Hz_, and ADC of conventional DWI) in different groups of AC.

Groups	d_mean_ (μm)	v_in_	d_ex_ (μm^2^/ms)	cellularity (×10^-2^)	ADC_PGSE_ (×10^-3^ mm^2^/s)	ADC_17Hz_ (×10^-3^ mm^2^/s)	ADC_33Hz_ (×10^-3^ mm^2^/s)	ADC (×10^-3^ mm^2^/s)
WHO grade								
Low-grade (n=39)	13.7929 ± 1.1411	0.2958 ± 0.0611	2.0936 ± 0.3096	2.4383 ± 0.5415	0.9023 ± 0.1526	1.2703 ± 0.1932	1.8963 (1.6249, 2.0579)	0.7518 ± 0.0848
High-grade (n=14)	13.8868 ± 1.8447	0.3096 ± 0.0680	2.1893 ± 0.3449	2.5124 ± 0.6100	0.8518 (0.7959, 0.9687)	1.2742 ± 0.1942	1.9381 (1.7199, 2.2843)	0.6850 ± 0.0833
*P* value	0.8604	0.4849	0.3404	0.6730	0.5860	0.9493	0.2672	0.0173
T stage								
pT1-2 (n=19)	13.8886 ± 0.9410	0.2940 ± 0.0696	2.1608 ± 0.3282	2.4074 ± 0.6361	0.9357 ± 0.1741	1.2996 ± 0.2407	1.9905 ± 0.2672	0.7600 ± 0.0986
pT3-4 (n=34)	13.7781 ± 1.5346	0.3025 ± 0.0593	2.0464 (1.9031, 2.2253)	2.4861 ± 0.5128	0.8794 ± 0.1475	1.2556 ± 0.1598	1.8315 (1.5995, 2.0734)	0.7197 ± 0.0806
*P* value	0.7769	1.5346	0.2209	0.6255	0.2183	0.4271	0.1282	0.1136
N stage								
pN0 (n=23)	13.4390 ± 1.2380	0.2904 ± 0.0741	2.1901 ± 0.3549	2.4182 ± 0.6255	0.9360 ± 0.2006	1.3038 ± 0.2241	1.9891 (1.6492, 2.2950)	0.7450 ± 0.09189
pN1-2 (n=30)	14.1080 ± 1.3678	0.3063 ± 0.0524	2.0643 ± 0.2819	2.4883 ± 0.5040	0.8717 ± 0.1120	1.2864 (1.1547, 1.3711)	1.8416 (1.6205, 1.6205)	0.7258 ± 0.0869
*P* value	0.0719	0.3654	0.1564	0.6534	0.1771	0.5418	0.2659	0.4410
PNI								
Negative (n=27)	13.8252 ± 1.2448	0.3051 ± 0.0699	2.0857 ± 0.2846	2.4928 ± 0.5823	0.8832 ± 0.1715	1.2609 ± 0.1864	1.8043 (1.6249, 2.1076)	0.7343 ± 0.0876
Positive (n=26)	13.8099 ± 1.4631	0.2935 ± 0.0548	2.1534 ± 0.3530	2.4216 ± 0.5349	0.9167 ± 0.1446	1.2823 ± 0.2000	1.9218 (1.7662, 2.1360)	0.7340 ± 0.0916
*P* value	0.9675	0.5077	0.4447	0.6454	0.4461	0.6880	0.2857	0.9929
LVI								
Negative (n=29)	13.5197 ± 1.2306	0.3013 ± 0.0702	2.1381 ± 0.3720	2.5024 ± 0.5760	0.9117 ± 0.1871	1.2702 ± 0.2276	1.9173 (1.6634, 2.2635)	0.7379 ± 0.9788
Positive (n=24)	14.1777 ± 1.4098	0.2972 ± 0.0533	2.0957 ± 0.2454	2.4041 ± 0.5364	0.8849 ± 0.1167	1.2727 ± 0.1411	1.8416 (1.6123, 1.9918)	0.7200 (0.6600, 0.7863)
*P* value	0.0756	0.8080	0.6215	0.5267	0.5271	0.9614	0.2455	0.7476
TB								
Negative (n=24)	13.2590 ± 1.3255	0.2873 ± 0.0669	2.0849 ± 0.3470	2.4609 ± 0.6031	0.9211 ± 0.1729	1.2645 ± 0.2153	1.9473 ± 0.3717	0.7423 ± 0.0959
Positive (n=29)	14.2800 ± 1.1908	0.3095 ± 0.0581	2.1470 ± 0.2965	2.4554 ± 0.5234	0.8818 ± 0.1457	1.2770 ± 0.1733	1.8963 (1.6481, 2.1138)	0.7274 ± 0.0834
*P* value	0.0048	0.2027	0.4851	0.9717	0.3730	0.8157	0.9857	0.5485

ADC, apparent diffusion coefficient; AC, adenocarcinoma; PNI, perineural invasion; LVI, lymphovascular invasion; TB, tumor budding.

### 3.5 Comparison of ROC curves

**Figure 5:**
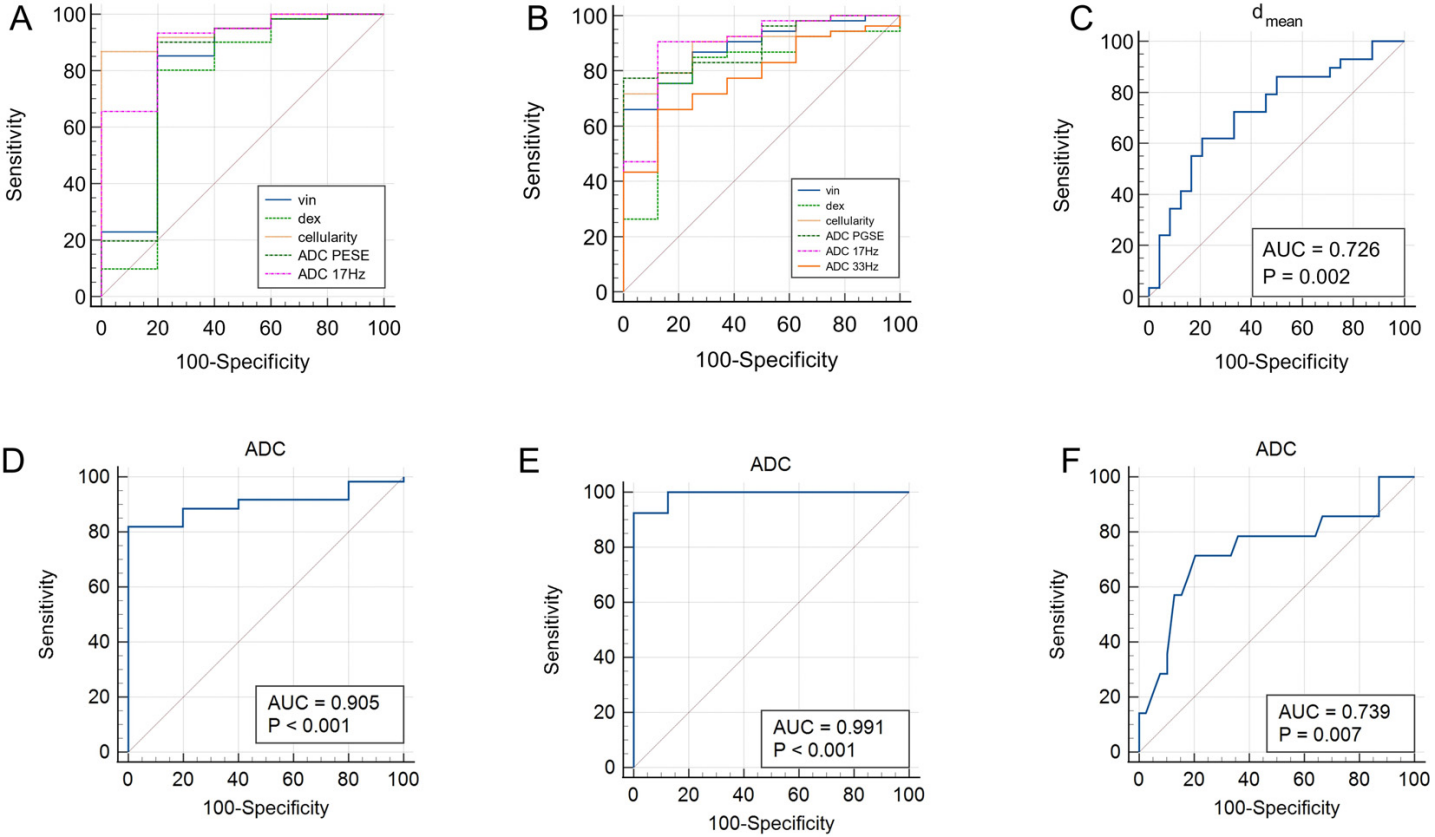
ROC curves of v_in_, d_ex_, cellularity and ADC values (ADC_PGSE_ and ADC_17Hz_) for discrimination between malignant and benign lesions **(A)**; ROC curves of v_in_, d_ex_, cellularity and ADC values (ADC_PGSE_, ADC_17Hz_, and ADC_33Hz_) for discrimination between AC and MC **(B)**; ROC curve of d_mean_ for discrimination between positive and negative TB **(C)**. ROC curves of ADC values (conventional DWI) for distinguishing malignant and benign lesions **(D)**, AC and MC **(E)**, low-grade and high-grade of AC **(F)**. ADC, apparent diffusion coefficient; AC, adenocarcinoma; MC, mucinous adenocarcinoma; TB, tumor budding.

The area under the curves (AUCs) for distinguishing malignant from benign lesions using the v_in_, d_ex_, cellularity, and ADC values (ADC_PGSE_, ADC_17Hz_, and ADC of conventional DWI) were 0.803, 0.757, 0.948, 0.807, 0.908 and 0.905,respectively (**Figures 5A, D**). The AUCs of v_in_, d_ex_, cellularity, and ADC values (ADC_PGSE_, ADC_17Hz_, ADC_33Hz_, and ADC of conventional DWI) for distinguishing AC from MC were 0.887, 0.802, 0.906, 0.896, 0.896, 0.781 and 0.991, respectively (**Figures 5B, E**). The AUC of the d_mean_ for evaluating TB status was 0.726 (**Figure 5C**). The AUC of ADC from conventional DWI for evaluating WHO grade was 0.739 (**Figure 5F**). The diagnostic performance and optimal diagnostic cut-off values were shown in **Table 6**.

**Table 6:** Diagnostic performance of d_mean_, v_in_, d_ex_, cellularity and ADC values (ADC_PGSE_, ADC_17Hz_, ADC_33Hz_, and ADC of conventional DWI) between different groups.

Category	Sensitivity	Specificity	AUC (95%CI)	Cutoff	*P* value
Malignant lesion vs. Benign lesion					
v_in_	85.25%	80.00%	0.803 (0.687-0.891)	0.2168	0.0392
d_ex_	80.33%	80.00%	0.757 (0.636-0.854)	2.4633 μm^2^/ms	0.1292
cellularity	86.89%	100.00%	0.948 (0.863-0.987)	1.8314×10^-2^	<0.0001
ADC_PGSE_	90.16%	80.00%	0.807 (0.691-0.893)	1.2017 (×10^-3^ mm^2^/s)	0.0473
ADC_17Hz_	93.44%	80.00%	0.908 (0.811-0.965)	1.6314 (×10^-3^ mm^2^/s)	<0.0001
ADC	81.97%	100%	0.905 (0.807-0.963)	0.90 (×10^-3^ mm^2^/s)	<0.0001
AC vs. MC					
v_in_	66.04%	100.00%	0.887 (0.780-0.954)	0.2720	<0.0001
d_ex_	75.47%	87.50%	0.802 (0.680-0.893)	2.3054 μm^2^/ms	0.0006
cellularity	71.70%	100.00%	0.906 (0.803-0.966)	2.081×10^-2^	<0.0001
ADC_PGSE_	77.36%	100.00%	0.896 (0.791-0.960)	0.9786 (×10^-3^ mm^2^/s)	<0.0001
ADC_17Hz_	90.57%	87.50%	0.896 (0.791-0.960)	1.5144 (×10^-3^ mm^2^/s)	<0.0001
ADC_33Hz_	66.04%	87.50%	0.781 (0.656-0.876)	1.9922 (×10^-3^ mm^2^/s)	0.0002
ADC	92.45%	100%	0.991 (0.924-1.000)	0.86 (×10^-3^ mm^2^/s)	<0.0001
Low-grade vs. High-grade					
ADC	71.43%	79.49%	0.739 (0.600-0.850)	0.68 (×10^-3^ mm^2^/s)	0.007
TB (+) vs. (-)					
d_mean_	62.07%	79.17%	0.726 (0.586-0.839)	14.0769 μm	0.002

ADC, apparent diffusion coefficient; AC, adenocarcinoma; MC, mucinous adenocarcinoma; TB, tumor budding.

## 4 Discussion

CRC showed an upward trend and have been one of the leading cancer types in China (12). Preoperative diagnosis of rectal cancer is extremely important for the prognosis of patients, especially the early diagnosis of benign and malignant rectal lesions. However, conventional MRI was unable to differentiate adenomas from adenocarcinomas demonstrated a potential risk for overstaging and consequently overtreatment (13). Jia et al. found that IVIM-DWI could predict rectal adenomas with canceration in a previous study (4), and the ADC value was significantly lower in the cancerous group than in the adenoma group. The recently developed td-dMRI technique has shown unique advantages in various diseases. A study by Lima et al. demonstrated that significantly lower ADC values were observed in malignant compared with benign head and neck tumors scanned using both OGSE and PGSE sequences (14). Ejima F et al. found that ADC_OGSE_/ADC_PGSE_ was significantly and strongly correlated with histological grade, FIGO stage, and prognostic risk classification in uterine endometrial cancer (15). In the present study, we investigated whether the ADC values derived from OGSE and PGSE sequences and the microstructural features quantified by the IMPULSED method could be helpful for the diagnosis of rectal lesion. The scan process went smoothly for all patients, and had no additional burden on patients. Our preliminary results illustrated that the v_in_, d_ex_, cellularity and ADC values can be used to differentiate malignant and benign lesions. In our study, the v_in_ and cellularity in malignant lesion were significantly higher, the d_ex_ and ADC values were significantly lower. The changes of ADC were in accordance with the reported literature. This may be related to the increase in the volume and nuclear cytoplasmic ratio of cancer cells, resulting the limitation of dispersion of intracellular and extracellular water molecules.

MC is a specific rectal cancer subtype, and it is defined by the World Health Organization (WHO) as an adenocarcinoma in which at least 50% of the extracellular space is occupied by mucin. In large population-based studies, the MC accounts for 10–15% of rectal cancer (16). MC is more prone to local recurrence (17) and metastasizes to lymph nodes (18). Therefore, it is important to differentiate MC from AC. However, distinguishing MC from AC by the subjective judgment of radiologists is difficult. Several new MRI techniques have been used to evaluate the pathological features of rectal cancer. Some studies found that the APT SI, T1 relaxation time, and ADC value of MC were significantly higher than those of AC (19, 20). Zhang et al. explored the value of T2 mapping and DWI in MC, and the results showed that patients with MC had higher T2 and ADC values than non-MC (3). Diffusion kurtosis imaging (DKI) was considered as a more valuable imaging biomarker than conventional DWI for differentiating MC from AC, and the AUCs of MK, MD and ADC for distinguishing MC from AC were 0.97, 0.95 and 0.88 (21). In our study cohort, the microstructural index of v_in_ and cellularity of rectal AC were significantly higher than MC, while d_ex_ and ADC values were lower in AC. We considered that these results were consistent with the component of MC, owing to its low cellular density and high amounts of water molecules, especially extracellular water. The AUCs for distinguishing AC from MC using v_in_, d_ex_, cellularity, ADC_PESE_, ADC_17Hz_, ADC_33Hz_ and ADC of conventional DWI were 0.887, 0.802, 0.906, 0.896, 0.896, 0.781 and 0.905, indicating high sensitivity and specificity.

The histological grade, tumor stage and lymph node metastasis are important factors in the selection of treatment plans and prognostic predictions for patients with rectal cancer. Some studies found that the ADC value by conventional DWI was not effective in determining T stages, N stages, or WHO grades of rectal cancer (5, 22). However, Ge et al. found that the mean T2 and ADC values for metastatic lymph nodes were significantly lower than for benign lymph nodes in patients with non-mucinous rectal adenocarcinoma (23). Chen et al. found that the APT SI was significantly higher in rectal tumors with high grade, T3 stage, and lymph node involvement (24). In our study, ADC value of conventional DWI was higher in low-grade group, and the AUC for evaluating WHO grade was 0.739. No significant differences in d_mean_, v_in_, d_ex_, cellularity, and other ADC values was observed in the subgroups of different histological grade, T and N stages, which may because the differences in tumor microenvironments were insufficient to cause significant changes or due to the limited sample size. In addition, metastatic lymph nodes were not evaluated separately in the present study, whether parameters from td-MRI can predict lymph node status needs more relevant research.

Recently, there is an increasing interest in PNI and LVI as a potential route of tumor spread, in addition to the well-known routes of direct extension, lymphatic metastasis, and hematogenous metastasis (25, 26). PNI is defined as the biological process characterized by cancer cells invading the nerves and spreading along the nerve sheaths. Recent advances in radiomics have been used to predict PNI status in rectal cancer (27–30), which demonstrating a good predictive effect, but the results lacked generalizability. Zhang et al. investigated the value of the pre-operative amide proton transfer-weighted (APTw) MRI to assess the prognostic factors in rectal adenocarcinoma, and they found PNI positive APTw signal intensities were higher than non-PNI group (31). LVI refers to tumor cells distributed in the endothelial lumen or destroying the corresponding lymphatic or vascular walls, which is considered a gateway to local spread or distant metastasis of cancer cells. Several studies using IVIM have been shown to be useful for in detecting LVI, but the diagnostic efficiency was limited (32, 33). We attempt to explore the value of IMPULSED MRI in predicting the status of PNI and LVI. However, there was no significant differences in positive and negative groups. The preoperative prediction of PNI and LVI status is still challenging and needs further study.

TB, as a process of epithelial-mesenchymal transition in tumors, refers to the presence of scattered tumor cells or small clusters of tumor cells with poor differentiation at the invasive front of the tumor, observed under high-power microscopy. TB was a strong negative prognostic predictor of survival in rectal cancer patients after neoadjuvant therapy (34). Qu et al. found that radiomics model based on MR T2WI could provide an effective and noninvasive method for preoperative TB grading assessment in patients with rectal cancer (35). Peng et al. assessed TB in rectal cancer using multiparameter MRI radiomics, and the AUC of the combined model was 0.961 and 0.891 in the training and validation cohorts, respectively (36). In our research, the d_mean_ showed high discriminative power in separating TB status in AC. The AUC of the d_mean_ for evaluating TB status was 0.726, indicating the potential value of the IMPULSED method for the noninvasive microstructural feature in rectal tumor.

However, the current study has some limitations. Firstly, this study was conducted at a single center and used a single MRI system. Secondly, our sample size was not large enough, which limited the diagnostic accuracy. Thirdly, we manually drew the region of interest (ROI), which may have a certain selection bias and cannot guarantee that necrotic and cystic tissues were completely free from ROI. Larger sample sizes and multicenter study are needed in the future to confirm the clinical value.

## 5 Conclusion

In summary, our study demonstrated that cellular microstructural mapping by the IMPULSED method were helpful in differentiating malignant and benign rectal lesions, and distinguishing AC from MC. The d_mean_ showed high discriminative power in predicting TB status of AC.

## Data Availability

The original contributions presented in the study are included in the article/supplementary material. Further inquiries can be directed to the corresponding author.
